# Uso juicioso de los smartphones en las consultas de atención primaria

**DOI:** 10.1016/j.aprim.2024.102975

**Published:** 2024-05-29

**Authors:** Raisa Maria Iconareasa Sugeac, Rosa Coll

**Affiliations:** a Facultat de Medicina, Universitat de Barcelona, Barcelona, España; b Servicio de Medicina Interna. Hospital Universitari Sagrat Cor. Quirón Salud, Barcelona, España

*Sr. Editor,*

He leído con interés el artículo titulado *Análisis de las interrupciones generadas por el uso de smartphones entre los profesionales sanitarios de Atención Primaria*^1^ que aborda un tema de gran importancia, que merece ser analizado con mayor profundidad para poder garantizar la excelencia en la atención médica. En la encuesta planteada por los autores, el 58,8%, de los pacientes tolera la interrupción a criterio del profesional y aumenta al 89% si es por algún aspecto relacionado con su visita.

La finalidad del uso de los móviles por parte de los profesionales es otra incertidumbre que los pacientes pueden tener, ya que parte de esas interrupciones pueden ser con fines sanitarios, consultando guías clínicas o protocolos^1^, lo cual plantea una dicotomía interesante entre la necesidad de recursos tecnológicos en la práctica médica y la percepción de los pacientes al respecto. Es decir, que si aceptan que su médico haga consultas sobre su caso, adecuadamente informados, deberían aceptar que recibiera una consulta de otro profesional sobre otro paciente.

Es innegable la importancia que ha tenido la digitalización sanitaria en los últimos años. Las aplicaciones sanitarias han ayudado a digitalizar contenido médico de cada paciente y facilitar el acceso a ello, tanto por parte de los profesionales como de los mismos pacientes^2^.

En el estudio comentado, el 10,1% de los pacientes sienten el uso de los móviles por profesionales sanitarios como una interrupción inadecuada durante el acto asistencial, lo que les genera un sentimiento de incomodidad o desconfianza si perciben que el médico está distraído o no completamente enfocado en su atención. Esta situación puede influir significativamente en la calidad de la relación médico-paciente. Los profesionales –según la misma encuesta– consultan su teléfono durante las visitas (92,4%), lo mantienen en silencio (77,7%), lo que les permite conocer la procedencia de la llamada y hacer un uso, a su criterio, adecuado (85,4%).

Estas herramientas tecnológicas también plantean dilemas éticos relacionados con la privacidad y la confidencialidad de los datos del paciente. Imaginémonos que 2 compañeros de la consulta se comuniquen por Whatsapp u otra red social sobre un paciente, ¿cómo nos aseguramos de que esa información permanezca confidencial? La necesidad de establecer protocolos claros y seguros para el manejo de la información sensible en entornos digitales es un aspecto que merece una atención especial.

En cuanto a los resultados del estudio, sería interesante analizar si la edad de los pacientes influye en su percepción de incomodidad respecto al uso de teléfonos móviles por parte de los profesionales sanitarios, debido a que puede haber diferencias generacionales en la percepción de estas prácticas. Asimismo, convendría saber las diferencias de los pacientes según distintos contextos culturales y socioeconómicos; las consecuencias que tiene el uso del móvil en los diferentes ámbitos sanitarios pueden ser distintas.

No es lo mismo usar el móvil en una consulta en atención primaria que dentro de un quirófano. El uso de móvil en un quirófano es una vía de transmisión de gérmenes y gran parte de los profesionales se lo llevan allí^3^, de forma que se debería plantear limitaciones del uso del móvil atendiendo a las diferentes especialidades.

Mencionan los autores la necesidad de establecer pautas y recomendaciones para el uso de smartphones en la atención primaria, que pueden derivar de la lectura del interesante artículo. Tenerlo en silencio, pedir permiso para utilizarlo (68,8% según los profesionales), uso profesional, no más de una interrupción por visita, duración breve de la interrupción.

## Conflicto de intereses

No hay fuente de financiación ni conflicto de intereses.
